# Act to Incorporate Veterinary Society

**Published:** 1885-04

**Authors:** 


### AN ACT TO INCORPORATE A SOCIETY FOR THE PROMOTION OF VETERINARY SCIENCE AND ART IN NEW JERSEY.

Sec. 1. *Be it enacted,* That any number of persons, not less than ten, who have been and are now connected with the "Veterinary Medical Association of New Jersey," desirous of promoting the interests of veterinary science and practice in this State, may associate themselves together for that purpose, adopt a corporate name and make a certificate in writing of their organization, with the names and residences of the persons making the certificate, and upon so doing shall be and are the "New Jersey State Veterinary Society."

SEC. 2. *Be it enacted,* That such society shall for its first year have the same officers as are now the officers of the "Veterinary Medical Association of New Jersey," shall adopt such Constitution and By-Laws and such rules and regulations as to its officers, its modes of business, and its conditions of membership, as a majority of all members of said society shall approve of.

SEC. 3. *Be it enacted,* That no person not at present a member of the "Veterinary Medical Association of New Jersey," shall become a member of the "New Jersey State Veterinary Society," unless he shall have received a veterinary or medical diploma or certificate from some incorporated medical or veterinary college or school, or have been examined by a Board of Examiners appointed by this Society, and declared competent for veterinary practice in this State; and said Society shall have full authority to judge of their admission, or of their continuance as members.

SEC. 4. *Be it enacted,* That this Society, hereby incorporated, shall have power to use a seal of its incorporation, and to own property to an amount not exceeding one thousand dollars, and in the name of its President and Secretary to sue and be sued.

